# Designing better frog call recognition models

**DOI:** 10.1002/ece3.2730

**Published:** 2017-03-30

**Authors:** Paul S. Crump, Jeff Houlahan

**Affiliations:** ^1^ Department of BiologyUniversity of New Brunswick‐Saint JohnSaint JohnNBCanada

**Keywords:** amphibian monitoring, automated acoustic monitoring, *Lithobates sylvaticus*, song scope, sound recognition models, transferability, type I and II errors, wood frogs

## Abstract

Advances in bioacoustic technology, such as the use of automatic recording devices, allow wildlife monitoring at large spatial scales. However, such technology can produce enormous amounts of audio data that must be processed and analyzed. One potential solution to this problem is the use of automated sound recognition tools, but we lack a general framework for developing and validating these tools. Recognizers are computer models of an animal sound assembled from “training data” (i.e., actual samples of vocalizations). The settings of variables used to create recognizers can impact performance, and the use of different settings can result in large differences in error rates that can be exploited for different monitoring objectives. We used Song Scope (Wildlife Acoustics Inc.) to build recognizers and vocalizations of the wood frog (*Lithobates sylvaticus*) to test how different settings and amounts of training data influence recognizer performance. Performance was evaluated using precision (the probability of a recognizer match being a true match) and sensitivity (the proportion of vocalizations detected) based on a receiver operating characteristic (ROC) curve‐determined score threshold. Evaluations were conducted using recordings not used to build the recognizer. Wood frog recognizer performance was sensitive to setting changes in four out of nine variables, and small improvements were achieved by using additional training data from different sites and from the same recording, but not from different recordings from the same site. Overall, the effect of changes to variable settings was much greater than the effect of increasing training data. Additionally, by testing the performance of the recognizer on vocalizations not used to build the recognizer, we discovered that Type I error rates appear idiosyncratic and do not recommend extrapolation from training to new data, whereas Type II errors showed more consistency and extrapolation can be justified. Optimizing variable settings on independent recordings led to a better match between recognizer performance and monitoring objectives. We provide general recommendations for application of this methodology with other species and make some suggestions for improvements.

## 1 Introduction

Acoustic surveys are commonly used to monitor the status or activity of animals that vocalize. Several groups of organisms, such as anuran amphibians, bats, birds, and marine mammals, are particularly suited to acoustic monitoring because of their dependence on vocalizations for major components of their life history including attracting mates, defending territories, and locating prey (Capp & Searcy, 1991; Kalko, 1995; Wells, 1977; Winn & Winn, 1978). Depending on the species and habitat, acoustic surveys can be more efficient at identifying vocalizing individuals to species, rather than attempting to observe the organism directly (Clark, Brown, & Corkeron, 2010; Heyer, Donnelly, McDiarmid, Hayek, & Foster, 1994). Knowledge about the vocal repertoire of a species can help us understand where the organisms occur (Weir, Fiske, & Royle, 2009), the conditions under which they perform certain behaviors (Klaus & Lougheed, 2013; Villanueva‐Rivera, Pijanowski, Doucette, & Pekin, 2011), as well as provide estimates of abundance (Borker et al., 2014; Buxton & Jones, 2012).

Traditionally, acoustic surveys have been conducted by humans present at the field site listening for vocalizations. Over the last few decades, however, the use of automated recording devices (ARDs) to assist or replace manual acoustic surveys has become more common (Digby, Towsey, Bell, & Teal, 2013; Hutto & Stutzman, 2009; Peterson & Dorcas, 1992; Venier, Holmes, Holborn, McIlwrick, & Brown, 2012). Whereas manual surveys are limited by the amount of time a human can be present at a field site, ARDs can be deployed and automatically record sound at remote locations for long periods of time on user‐defined schedules (Acevedo & Villanueva‐Rivera, 2006). The main advantage in the use of ARDs over manual surveys is the increase in the amount and scope of environmental recordings and, therefore, an increase in the likelihood of detecting a species if it is present at the site (i.e., the detection probability). The probability of detecting a species has been shown to vary by, among others things, the time of year, time of the day, temperature, humidity, and abundance (Jackson, Weckerly, Swannack, & Forstner, 2006; Tanadini & Schmidt, 2011; Weir, Royle, Nanjappa, & Jung, 2005). If surveys are conducted when detection probabilities are low, the species could be missed when it is actually present.

Concern about the consequences of incorrectly concluding that a species is absent from a site (i.e., a false negative) is a topic of considerable interest in ecology (MacKenzie et al., 2006). It has been documented that estimating site occupancy without controlling for detection probability can result in a negative bias in the estimate of the occupied area (MacKenzie et al., 2002) as well as biased extinction and colonization rates (MacKenzie, Nichols, Hines, Knutson, & Franklin, 2003), and species distribution models (Comte & Grenouillet, 2013). Automated recording devices can help alleviate the problem of low detection probabilities and therefore increase the usefulness of survey data, by rapidly increasing the cumulative detection probability because of the additional listening time. For example, if the probability of detecting a rare frog during a five‐minute acoustic survey is 0.2, then a single manual survey at any site will detect the species when it is present about 20% of the time. With an ARD deployed at the site with a recording schedule of five minutes every 30 min from 7 p.m. to 7 a.m., the 25 recordings will yield a cumulative detection probability of .996 (using the equation 1 − (1 − *p*)^*N*^ where *p* is the detection probability and *N* is the number of surveys). However, this only means there is a good chance that if the species is present it was recorded—it must still be detected on the recording.

The quantity of samples generated by ARDs is often overwhelming. With the deployment of just five ARDs on a standard recording schedule, the recordings generated during a week of deployment easily exceed the hours of a typical work week. Several methods have been suggested to extract the required information from the large quantity of recordings. One approach to processing large amounts of recorded data is to use automated sound recognition algorithms that allow researchers to search all the recordings with a custom‐built model of the vocalization of interest (Acevedo, Corrada‐Bravo, Corrada‐Bravo, Villanueva‐Rivera, & Aide, 2009; Brandes, 2008). The goal of automated sound recognition is to identify the vocalization of a target species within the recordings among the other animal and environmental noises. Software programs can batch process hundreds of digital recording files, saving tremendous amounts of time in extracting information from the recordings (Waddle, Thigpen, & Glorioso, 2009; Willacy, Mahony, & Newell, 2015).

The vast quantity of acoustic samples obtainable from the deployment of ARDs coupled with the automated analysis of the recordings is a powerful tool for developing robust estimates of occupancy, extinction and colonization rates, and activity/phenology patterns. Several off‐the‐shelf software programs are available for researchers to conduct automated analysis of sound files for vocalizations of interest. However, the lack of published research utilizing these tools to answer questions at large scales hints at the difficulty in extracting information from the acoustic samples (Swiston & Mennill, 2009), and/or the reluctance of ecologists and wildlife managers to trust results from a fully automated process.

**Figure 1:**
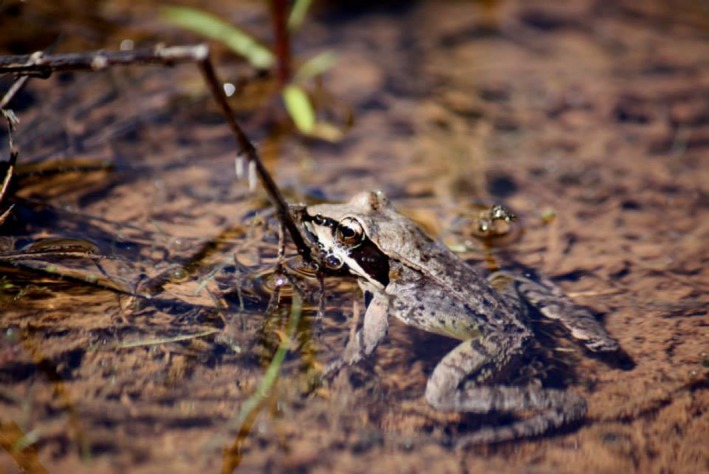
Photograph of the study subject, an adult male wood frog (*Lithobates sylvaticus*) (Photograph taken by R. Rommel‐Crump)

We used the software program Song Scope V 4.1.3A (Wildlife Acoustics, Concord, MA, USA) in our study. Song Scope is a commercially available, multi‐purpose sound analysis software program that has been used by ecologists to develop automated vocalization recognition models, or “recognizers” (Buxton & Jones, 2012; Holmes, McIlwrick, & Venier, 2014; Waddle et al., 2009; Willacy et al., 2015). Developing recognizers in Song Scope involves two steps. The first step is locating vocalizations from existing recordings (i.e., “annotating” the recordings) to be used as training data upon which the model is to be based. The second step is selecting the settings of the variables used to create the recognizer model. At the first step, we need to answer questions about how much, and what kinds of training data provide the best recognizers. At the second step, we need to identify the variable settings that build the best model (i.e., low false‐positive rates, low false‐negative rates, and good discriminatory ability). The manufacturers of Song Scope provide a general overview of and recommendations for the creation of recognizer models, but deciding on the quantity of training data and settings of the variables for model creation is a largely trial‐and‐error procedure, and we have found no published guidance. Their process emphasizes model performance on the training data (rather than new data where it will invariably be used). The primary purpose of this article is to provide guidance for designing and validating recognizers. More specifically, we asked (1) how does increasing training data influence recognizer performance and does the source of the training data matter, (2) is there an objective and repeatable way to choose variable settings and design a recognizer, which explicitly considers Type I and II errors in the process, and (3) can we extrapolate recognizer performance from the training dataset so that we can use it on new data with any degree of confidence? We use vocalizations of the wood frog (*Lithobates sylvaticus*) for all our experiments. Wood frogs are a common, North American pond‐breeding anuran and are a model organism for research into wetland ecosystem structure, amphibian population, and community ecology (Figure 1).

## 2 Methods

### 2.1 Building recognizers

#### 2.1.1 Song Scope software

Song Scope uses hidden Markov models (HMMs) to construct a model of the vocalization of interest from training data and compares this model to candidate vocalizations from the recordings. Interested readers should consult with Agranat (2009) for the technical specifics of the algorithm.

#### 2.1.2 Recognizer development

**Figure 2:**
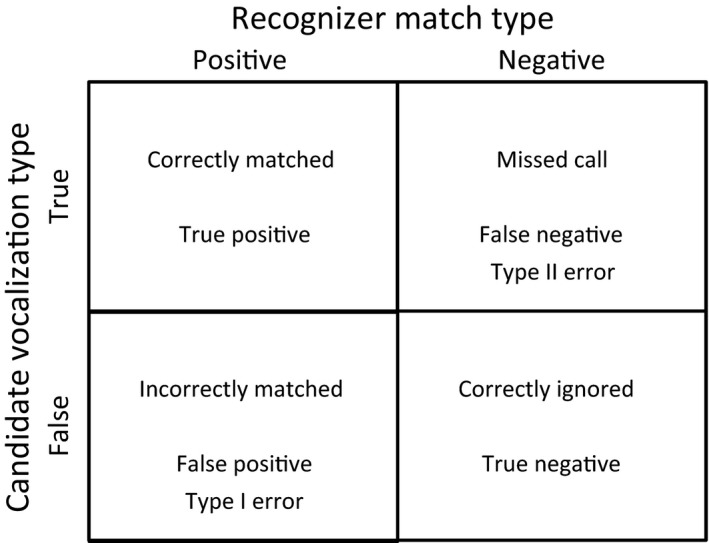
Confusion matrix showing the four possible outcomes of a recognizer match on a candidate vocalization

Recognition of the target vocalization is accomplished in a two‐step process. The first is detection, during which the recognizer model scans all sounds within the recording to identify the sounds that are potential target vocalizations. We define “sound” as any signal or noise in the recording—it may or may not be the target call. We use “vocalization” and “call” synonymously to refer to the true signal of the target species and “match” or “hit” when the recognizer model identifies a sound. Identifying the target vocalizations is done by comparing sounds to a model created by the program from annotated calls provided by the user. Signals are detected if they stand out against background noise and have roughly the same frequency range and temporal properties (call length, syllable structure, etc.) as the model. The second part involves computing a “score” statistic on sounds identified as potential target vocalizations at the detection step. This is a measure of similarity between the sound and the model (the similarity score can vary from 0 to 100, with 100 being a perfect match) and is generated by the Viterbi algorithm (Agranat, 2009). When the model encounters a sound, one of four outcomes occurs—true positive, false positive, true negative, or false negative (Figure 2).

True and false positives can be estimated by manually verifying the matches in the output, and false negatives can be determined by subtracting the number of true positives from the total number of vocalizations in the recording. True negatives are sounds that are not calls and not misidentified as calls.

The objective of recognizer development is to minimize the number of false positives and false negatives. There is an inevitable trade‐off between false positives and false negatives because to reduce false positives we must set a threshold so that only high‐quality vocalizations are matched, and many lower‐quality vocalizations are ignored. When reducing false negatives, the threshold for concluding a sound is a call is lower, and therefore, many lower‐quality sounds are included. Score values can be used to distinguish between true‐ and false‐positive matches. Ideally a threshold should be established, above which the match is certain to be a true positive, and below which the match is certain to be a false positive. However, this is rarely attained in practice, and the objective is to set a threshold that results in large reductions in Type I errors with only small increases in Type II errors.

#### 2.1.3 Recognizer metrics

In the following experiments, we use precision and sensitivity as our common metrics of recognizer performance. Precision is also known as the positive predictive value in signal detection theory (Fawcett, 2006) and is calculated as the number of true positives divided by the total number of recognizer matches. Precision provides an estimate of the probability of the recognizer match actually being the target vocalization, and (1‐precision) is equal to the Type I error or false discovery rate. Sensitivity, also known as true‐positive rate or recall, is calculated as the total number of true positives divided by the total number of true calls, whether detected (true positives) or not (false negatives). Sensitivity provides an estimate of the proportion of vocalizations detected by the recognizer, and (1‐sensitivity) is equal to the Type II error or false‐negative rate. Sensitivity is also an estimate of the detection probability (*p*) commonly used in occupancy modeling (Miller et al., 2012). We conditioned the estimates of precision and sensitivity on an optimal score threshold, determined using the area under a receiver operating characteristic (ROC) curve. The optimum threshold for each recognizer was determined using Youden's J statistic (Youden, 1950), where J = sensitivity + true‐negative rate – 1. We used the term “conditional” when referring to precision and sensitivity derived using the ROC threshold because if a different threshold was used, the precision and sensitivity would change. We estimated the “conditional” precision as the number of true‐positive matches above the ROC‐determined threshold divided by the total number of matches above the threshold (i.e., 1‐precision at the optimal score threshold = the Type I error rate). Similarly, the “conditional” sensitivity is estimated by the number of true‐positive matches above the threshold divided by the total number of calls in the recording (i.e., 1‐sensitivity at the optimal score threshold = the Type II error rate).

### 2.2 Improving recognizer performance

#### 2.2.1 Increasing training data

We assessed the effect of increasing training data on the identification of wood frog vocalizations. We started by collecting training data by annotating wood frog vocalizations from 28 sites in southern New Brunswick, Canada, recorded in 2012. The recordings from which the annotations were extracted were collected as part of a monitoring program and were recorded by Song Meter 1, SM2, and SM2 + units (Wildlife Acoustics, Concord, MA, USA). We annotated a total of 4,080 wood frog vocalizations with the primary objective to determine what training data to use to create a good recognizer. We made the assumption that there is variability in wood frog vocalizations among individuals, and this variability potentially affects the performance of the recognizer. There are three different levels across which training data can be collected and thus variability in vocalizations captured. These are (1) within recordings (i.e., same five‐minute recording at same site), (2) among recordings (i.e., different five‐minute recordings at same site), and (3) among sites. There should be more variability in vocalizations among different sites, as they will all be different individuals, than within a recording, as they are likely to be the same individuals.

For within‐recording variability, we used 1, 2, 4, 5, 6, 7, 8, 10, 11, and 12 calls (with number of recordings held at 15 and number of sites held at 28). For among‐recording variability, we used 1, 2, 4, 6, 8, 9, 10, 11, 13, 14, and 15 recordings from each site (with number of calls per recording held at 12 and number of sites held at 28). For among‐site variability, we used 1, 2, 5, 8, 11, 14, 17, 20, 23, 25, and 28 sites (with number of calls per recording held at 12 and number of calls per site held at 15). This allowed us to examine which sources of variability had the largest impact on recognizer performance. This initial approach resulted in 32 recognizer models. We then created another 11 recognizers to explore interactions among sources of variability, among‐site (*n* = 3), among‐recording (*n* = 6), and within‐recording (*n* = 2) training data. This resulted in a total of 43 recognizer models. Occasionally, we were unable to find as many calls as we had targeted so, the exact number of calls per recording and recordings per site used in the recognizers had some variability, and the total number of calls was often lower than our target. We report the average achieved number of calls per recording and recordings per site. The details of the targeted and achieved training data sources and totals for each recognizer can be found in Appendix S1.

Each recognizer was tested on 40 different five‐minute recordings (datasets Train and A–D, Table 1), and we manually reviewed all matches. To estimate the effect of increasing the total and type of training data on the recognizer performance, we used beta regressions (because precision and sensitivity are values between zero and one) and a logit link function. The mean conditional precision and mean conditional sensitivity across the 40 recordings were dependent variables, and the amount and type of training data were independent variables. We used Akaike's information criterion (AICc) adjusted for small sample sizes for model selection (Burnham & Anderson, 2002). Analysis was done in R 3.1.3 using the packages betareg (Cribari‐Neto & Zeileis, 2010) and AICcmodavg (Mazerolle, 2015). Plots were created using ggplot2 (Hadley, 2009).

**Table 1:** List of the characteristics of the training and test datasets used in recognizer evaluation. Values reported in the table are means from the recordings within the dataset. SNR is signal‐to‐noise ratio. Each dataset is comprised of eight five‐minute recordings; all recording sets have five recordings with wood frog calls and three without. Test set A has the same sites as used to build the recognizer and select the variable settings but from a different year. Test set B has different sites but from the same year. Test set C is from different sites and a different year. Test set D contains recordings from outside the study area in the USA, specifically the states Connecticut, Massachusetts, Michigan, and New York

Dataset	Sites	Year	*N* calls	SNR (dB)	Noise (dB)	Noise *SD* (dB)	Air temperature (°C)
Train	1, 2, 3, 12, 14, 29, 30, 35	2013	359	13.29	63.06	7.29	9.4
A	3, 14, 24, 29, 30, 34, 37, 50	2014	725	5.07	69.23	4.18	6.0
B	5, 19, 20, 27, 33, 41, 46, 100	2013	660	5.83	74.13	2.4	6.8
C	6, 17, 23, 28 38, 45, 49, 52	2014	181	3.35	77.42	4.86	4.3
D	USA	2015	571	4.93	83.02	4.96	11.6

#### 2.2.2 Variable sensitivity analysis

We assessed the effect of changes in the variable settings of recognizer models on the identification of wood frog vocalizations. Song Scope V.4.1.3a contains 11 different variables that are used to build recognizers that can be manipulated by the user. We excluded maximum and minimum frequencies of the bandwidth filter from the sensitivity analysis because of the consistent frequency range of most animal signals, and unlike other settings, the frequency range can be easily determined beforehand. Settings of the remaining nine different variables range from a few discrete choices to a large range of continuous values (Appendix S2). The number of variables combined with the number of possible settings for each variable presents some challenges in recognizer design. With most variables, there is no intuitive or empirical way to decide on the setting a priori; thus, to determine the optimal settings of the recognizer model, each variable needs to be evaluated for its effect on error rates.

We employed a local optimization method where each of the nine variables was evaluated independently across the range of possible values for that variable. All other variable settings were held at constant values. Examining interactions among variables was impractical because of the large number of possible combinations. As it was we evaluated, a total of 75 different recognizer models (Appendix S2) and a total of 255,966 recognizer matches were manually verified. The preliminary recognizer model was constructed using 936 wood frog vocalizations from five sites in southern New Brunswick recorded in 2012 and default variable settings. We created a “training” set of eight five‐minute recordings from different sites in 2013 to examine the effect of changes to variable settings in a standardized way. The recordings were manually inspected to estimate the number of wood frog vocalizations. We estimated the signal‐to‐noise ratio by randomly selecting two groups of 10 one‐second segments, one group with wood frog calls in them (signal + noise) and the other without wood frog calls (noise only). We measured the dB level and subtracted the mean of the noise only dB measurements from the mean of the signal + noise dB measurements to estimate the signal‐to‐noise ratio (Table 1). We used the mean conditional precision and conditional sensitivity from these recordings in the dataset to evaluate recognizer performance.

After each recognizer was built and the training file set scanned, we manually reviewed all the matches. A subset of the recordings was reviewed by two people so that observer error could be estimated. The observer error rate was estimated to be 0.1%. We used the coefficient of variation (CV = standard deviation/mean) of the conditional precision and conditional sensitivity to evaluate changes in the variables settings. The size of the CV is positively related to the sensitivity of recognizer metrics to changes in the variable setting.

Due to the multiple, almost identical, syllables in wood frog vocalizations, some variable settings resulted in conditional sensitivity values exceeding one, indicating that the recognizers were making multiple “true‐positive” matches by matching multiple syllables in a single true call. Using these high sensitivity values to select variables would change the focus of the recognizer to syllables, rather than calls. However, some true wood frog calls are single syllable calls so there is no single “correct” call type to model. To attempt to penalize these variable settings for making excess true matches, instead of concluding these multi‐syllable matches were false positives (which technically they are not) we randomly sampled *N* true‐positive matches without replacement, where *N* equals the number of real calls in the recording, and recalculated the precision and sensitivity using a subset of the true positives. For example, if there were 1,000 true calls but 1,500 true positives (i.e., the recognizer matched the two different syllables in 500 true calls), we sampled *N* = 1,000 of the true‐positive matches, scaling sensitivity between zero and one. We repeated this 1,000 times for each recording and used the mean of these randomizations as the conditional precision and conditional sensitivity values for the variable setting selection process.

The two criteria (conditional precision and conditional sensitivity) used to evaluate recognizers are directly linked to Type I and II errors (Type I error rate = 1‐precision and Type II error rate = 1‐sensitivity) and thus are often inversely related. Due to this relationship, there is no single “best” recognizer, the choice of whether to minimize Type I or II errors should depend on the objectives of the monitoring program. To assess how assumptions about the relative costs of Type I and II errors affected recognizer optimization, we evaluated overall recognizer performance using a weighted average linked to the relative cost of the errors. The weights for Type I and II errors were varied to simulate different monitoring objectives. The variable settings with the lowest weighted average error rate were then selected as the optimal setting for that recognizer. Value judgments are inherent in deciding relative costs, but by objectively and explicitly stating the goals of the monitoring project and linking those goals to the weights placed on the different recognizer parameters it is possible to increase both transparency and repeatability. Three final recognizers were created based on varied error weights (equal weight for Type I and II errors, weight of Type I errors 5× greater than Type II, and the weight of Type II errors 5× greater than Type I), and the training dataset was then reassessed to examine final model performance. For comparison, we also assessed a recognizer model that was developed by the more conventional trial‐and‐error approach where the best variable settings were chosen based on the training data used in the model. We termed this the “original” recognizer. The effort invested in evaluating all the variable settings was substantial, and we wanted to compare a labor‐intensive approach with a “quick‐and‐dirty” approach (i.e., the “original” recognizer) to see whether the extra effort was warranted. We report mean error rates and bootstrapped 95% confidence intervals of the means.

All data analyses were done in R.3.1.3 (R Core Team 2015). The area under the receiver operating characteristic curve (AUROCC) was determined using the pROC package (Robin et al., 2011). Bootstrapped confidence intervals (Bias‐corrected and accelerated) were calculated using the boot package (Canty & Ripley, 2015). Plots were created using ggplot2 (Hadley, 2009).

### 2.3 Evaluating recognizer performance

The primary goal in using recognizers is to accurately identify vocalizations at new sites and times. The acoustic characteristics of anuran calls have been shown to vary within a species among years (Wagner & Sullivan, 1995) and systematically among populations (Wycherley, Doran, & Beebee, 2002). In addition, it is of interest to know how well a recognizer created in one place/time performs at others to know whether centralized automated monitoring programs are feasible. We compared the three recognizers built in part 2 above and optimized on independent training data with a recognizer built using the more conventional approach of maximizing the fit to the training data used for calls in the model. To explore how the performance of these four recognizers varied, we used them on four additional datasets not used in recognizer creation (Table 1). We used the score thresholds determined from the training data to make the predictions. The four datasets were created by randomly choosing files (A) from sites used either to collect training data or to select variable settings, but from a different year, (B) from sites not used for training data or variable setting selection but from within the study area and from the same year, (C) from different sites and years than used for the training data or variable setting selection but from within the study area, and (D) from sites outside the study area (US states Connecticut, Massachusetts, Michigan, and New York) and from a different year.

## 3 Results

### 3.1 Increasing training data

**Figure 3:**
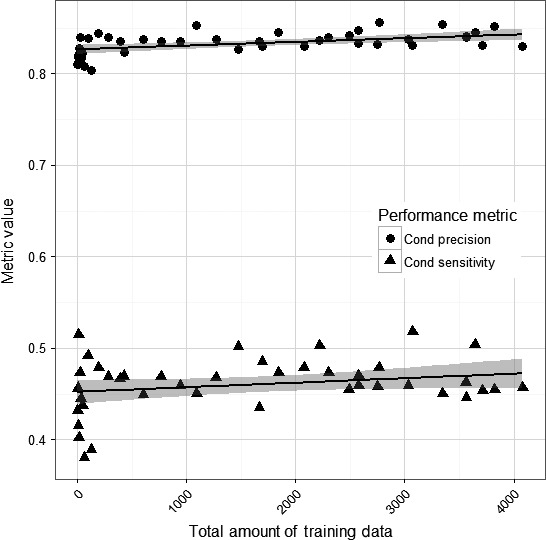
The effect of increasing the total amount of training data (number of calls) on the conditional precision (1—Type I error rate, pseudo‐*R* ^2^ = .2238, untransformed logit coefficient 0.00002872 ± *SE* 0.000008085) and conditional sensitivity (1—Type II error rate, pseudo‐*R* ^2^ = .06327, untransformed logit coefficient 0.00002014 ± *SE* 0.00001182)

To evaluate the three different ways of increasing training data, a total of 385,452 recognizer matches were manually verified. Overall, irrespective of the source, increasing annotations from a minimum of four vocalizations (one call from each of four recordings at the same site) to a maximum of 4,080 (10.3 calls from 13.5 recordings at 28 sites) resulted in only small improvements to the mean conditional precision and conditional sensitivity (Figure 3). The fitted values from the full model for conditional precision increased from 0.817 with four calls used for training data to 0.839 with 4,080 calls (i.e., increasing the number of training calls by three orders of magnitude improved the precision of the recognizer by ~2%). The fitted values from the full model for conditional sensitivity increased from 0.435 with four calls for training data to 0.479 with 4,080 calls (similarly, the sensitivity increased by ~3%).

**Figure 4:**
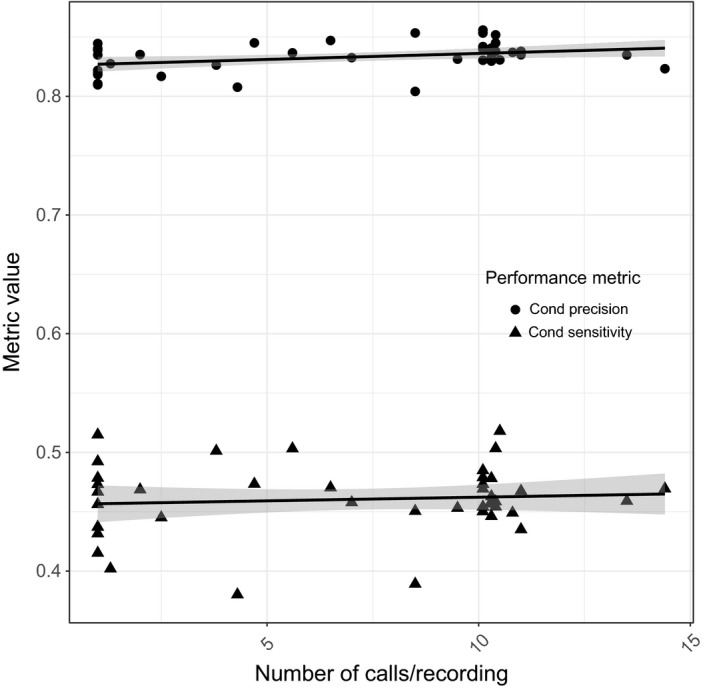
The effects of increasing the number of calls from each recording (within recordings) used to build the recognizer model on the conditional precision (1—Type I error rate, pseudo‐*R* ^2^ = .1212, untransformed logit coefficient 0.007175 ± *SE* 0.002922) and conditional sensitivity (1—Type II error rate, pseudo‐*R* ^2^ = .008546, untransformed logit coefficient 0.002538 ± *SE* 0.004162)

**Figure 5:**
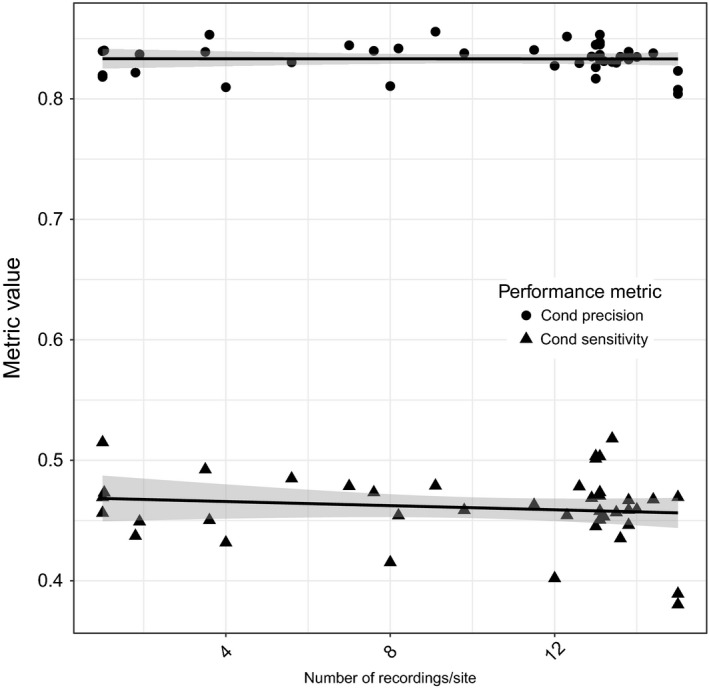
The effects of increasing the number of recording files (among recordings) from each site used to build the recognizer model on) the conditional precision (1—Type I error rate, pseudo‐*R* ^2^ > .001, untransformed logit coefficient −0.0001286 ± *SE* 0.0028096) and conditional sensitivity (1—Type II error rate, pseudo‐*R* ^2^ = .02011, untransformed logit coefficient −0.003491 ± *SE* 0.003712)

**Figure 6:**
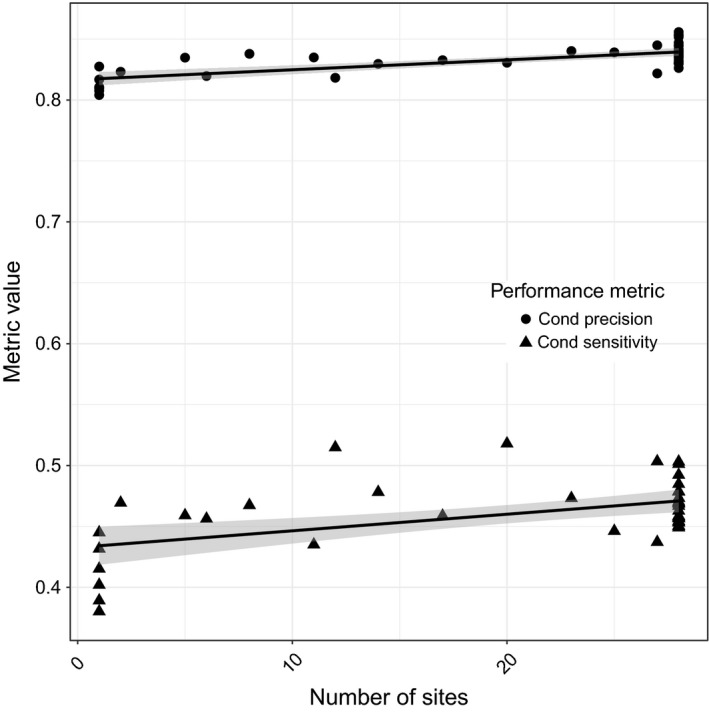
The effects of increasing the number of sites (among sites) used to build the recognizer model on the conditional precision (1—Type I error rate, pseudo‐*R* ^2^ = .5012, untransformed logit coefficient 0.0057268 ± *SE* 0.0008514) and conditional sensitivity (1—Type II error rate, pseudo‐*R* ^2^ = .2623, untransformed logit coefficient 0.005522 ± *SE* 0.001418)

However, the effects of increasing the quantity of within‐recording training data (Figure 4), increasing among‐recording training data (Figure 5), and increasing among‐site training data (Figure 6) were different. Increasing training data among sites and within recordings had a positive effect on conditional precision (Table 2A). For conditional sensitivity, only the number of sites had a positive effect (Table 2B). The effect of among‐recording variation on both conditional precision and conditional sensitivity was negative, but the 95% confidence interval overlapped zero. The model including only among‐site variation in training calls had the most support, but unexpectedly the full model, including within‐recording, among‐recording and among‐site variation training calls, had almost as much support (Table 3). The beta distributions were examined separately and had the following alpha and beta parameters for CPPV, α = 8.63, β = 1.73, and CTPR, α = 3.50, β = 4.10.

**Table 2:** Beta‐distributed generalized linear models with A) conditional precision and B) sensitivity as the dependent variable. All intercepts, coefficients, and standard errors are in their untransformed logit linked state

Model	Intercept	*SE*	Sites	*SE*	Recordings	*SE*	Calls	*SE*
(A) Conditional precision								
Sites	1.493	0.02	0.006	0.0008	–	–	–	–
All sources	1.469	0.03	0.005	0.0008	−0.0004	0.002	0.005	0.002
(B) Conditional sensitivity								
Sites	−0.271	0.03	0.006	0.001	–	–	–	–
All sources	−0.249	0.05	0.005	0.001	−0.002	0.003	0.001	0.003

**Table 3:** Model selection results for beta regressions with A) conditional precision and B) sensitivity as the dependent variable. The total calls model represents the situation where increasing training data results in better recognizer performance irrespective of the source of the training data. Evidence ratios for model comparisons are calculated by dividing the model weights. A = among sites and recordings and W = within recordings

Model	*K*	AICc	∆ AICc	Model weight	Log likelihood	Pseudo‐*R* ^2^
**(A) Conditional precision**						
A/sites	3	−282.11	0	0.51	144.36	.502
All sources	5	−282.03	0.08	0.49	146.83	.554
Total calls	3	−262.76	19.35	0	134.69	.224
W/recording	3	−257.37	24.74	0	131.99	.121
Intercept only	2	−254.11	28.00	0	129.20	–
A/recordings	3	−251.80	30.32	0	129.21	>.001
**(B) Conditional sensitivity**						
A/sites	3	−189.99	0	0.89	98.30	.262
All sources	5	−185.48	4.50	0.09	98.55	.271
Total calls	3	−179.74	10.25	0.01	93.18	.063
Intercept only	2	−179.24	10.75	0	91.77	–
A/recording	3	−177.80	12.19	0	92.21	.020
W/recording	3	−177.30	12.69	0	91.96	.009

The small difference in AICc and log likelihood values between the top models for conditional precision indicates that among‐site variability is driving the relationship, but there was a minor role for additional training data from within recordings. For conditional sensitivity, the effect of among‐site training data was clear as seen by the change in AIC between the top models. In summary, the most efficient way to increase recognizer performance was to include vocalizations from more sites in the recognizer model, but even this effect was weak.

### 3.2 Variable sensitivity analysis

**Figure 7:**
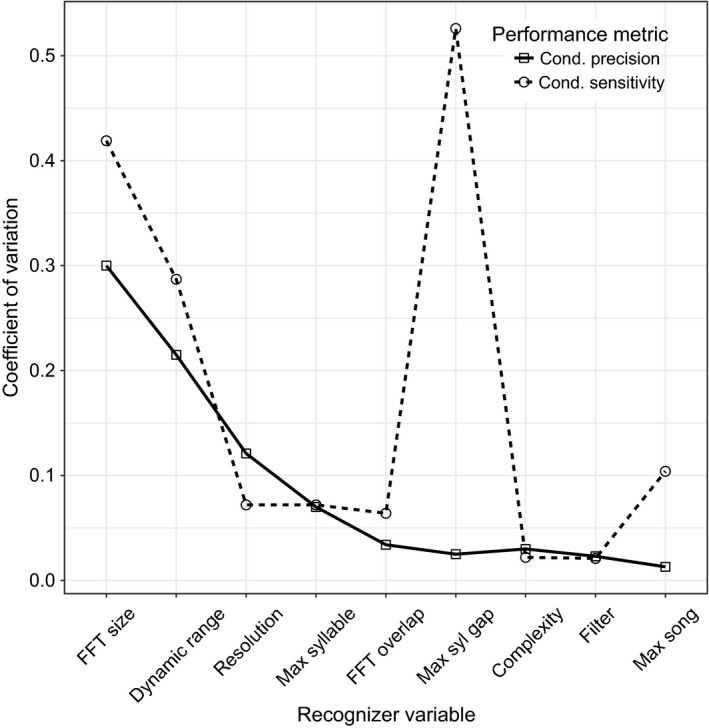
The rank sensitivity of the conditional precision and sensitivity of the nine recognizer variables plotted in order of descending coefficient of variation (CV) values for conditional precision

All nine variables affected the performance of the recognizers, but their effects differed. The conditional precision of the recognizer was most sensitive to changes in fast Fourier transform (FFT) size (CV = 0.3), dynamic range (CV = 0.215), and resolution (CV = 0.121) (CV equal to or greater than 0.1). The conditional sensitivity was affected most by changes in maximum syllable gap (CV = 0.526), FFT size (CV = 0.419), dynamic range (CV = 0.287), and maximum song length (CV = 0.104) (see Appendix S2 for a description of the variables). Figure 7 shows rank sensitivity curves of the all the variables against the CV of the conditional precision and conditional sensitivity. Overall, conditional sensitivity was more affected (CV = 0.179) by changes in variable settings than conditional precision (CV = 0.133).

**Figure 8:**
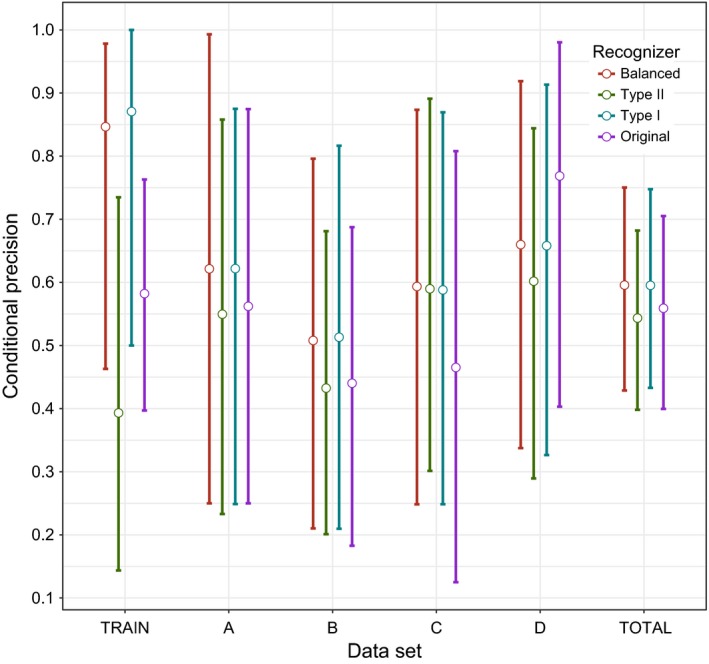
The mean (open circles) and bootstrapped 95% confidence intervals (error bars) of precision (1—Type I error rate) for the four final recognizer models. The balanced recognizer is the recognizer where Type I and II errors are weighted equally, Type II is the recognizer designed to minimize Type II errors, Type I is the recognizer designed to minimize Type I errors, and original is the recognizer designed using the trial‐and‐error approach. The “Train” dataset is the recordings used to select the variable settings. Test set A used the same sites to build the recognizer and select the variable settings but from a different year. Test set B has different sites but from the same year. Test set C is from different sites and a different year. Test set D contains recordings from outside the study area in the USA recorded in 2015, specifically the states Connecticut, Massachusetts, Michigan, and New York (see Table 1). The “Total” dataset is the combined datasets A–D

**Figure 9:**
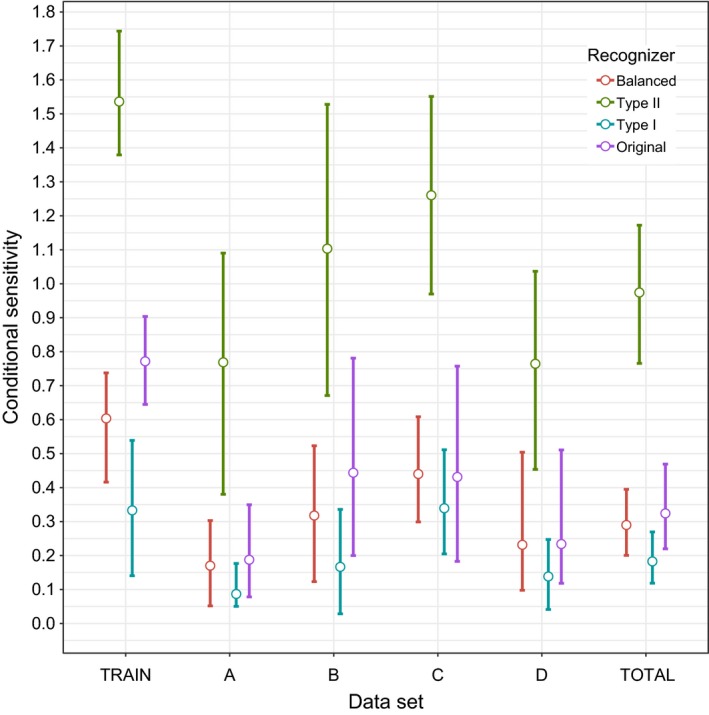
The mean (open circles) and bootstrapped 95% confidence intervals (error bars) of sensitivity (1—Type II error rate) for the four final recognizer models. The balanced recognizer is the recognizer where Type I and II errors are weighted equally, Type II is the recognizer designed to minimize Type II errors, Type I is the recognizer designed to minimize Type I errors, and original is the recognizer designed using the trial‐and‐error approach. The “Train” dataset is the recordings used to select the variable settings. Test set A used the same sites to build the recognizer and select the variable settings but from a different year. Test set B has different sites but from the same year. Test set C is from different sites and a different year. Test set D contains recordings from outside the study area in the USA recorded in 2015, specifically the states Connecticut, Massachusetts, Michigan, and New York (see Table 1). The “Total” dataset is the combined datasets A–D.

Final recognizers performed according to the weights placed on the errors when the training dataset was reassessed, but there was considerable overlap in confidence intervals (Figures 8 and 9). The Type I recognizer had the highest mean precision of 0.87 (CI 0.5 – 1) closely followed by the balanced recognizer at 0.85 (CI 0.46–0.98). The Type II recognizer had the highest sensitivity (mean 1.54, CI 1.35–1.74), and despite our efforts to impose a penalty for this at the setting selection stage, it overestimated the number of real calls by making separate hits on different syllables of the same call (i.e., we did not “correct” the results of the recognizer to reduce the sensitivity to below 1; we attempted to prevent this from occurring at the variable selection stage but failed). The ranks of the recognizer models for conditional sensitivity indicate that selecting variable settings empirically can lead to reductions in the Type II errors beyond that of using a trial‐and‐error approach on the training data. This was not the case for conditional precision.

### 3.3 Evaluating recognizer performance

Precision (i.e., reduced Type I errors) varied across the test datasets A–D but showed only small differences among recognizers (Figure 8). Not surprisingly, the recognizer designed to maximize sensitivity (i.e., reduced Type II errors) had the highest sensitivity across all four datasets (Figure 9). Confidence intervals extending well above 1 again show the propensity of the recognizer to overestimate calls is not limited to the training data. There was little consistency in recognizer performance across datasets, and mean error rates were generally higher when recognizers were applied to new data. The errors were not related in any obvious way to the differences in sites and times between the training set and test datasets. In fact, the highest precision occurred at the sites furthest away from the sites the recognizer was developed from (dataset D, Figure 8).

## 4 Discussion

Our objective here was to improve the utility of sound recognition tools for surveying vocalizing anurans and to try to remove some of the barriers to widespread use in ecology. The need to monitor biological diversity at large spatial and temporal scales is becoming increasingly important (Yoccoz, Nichols, & Boulinier, 2001). While citizen science (Weir et al., 2009) and manual surveys by professional biologists are widely used, fully automated platforms using ARDs and sound recognition software could help to meet new monitoring challenges. We examined three critical components in sound recognition (training data, variable setting selection, and prediction to new data), and this provides guidance for the future use of recognizers for monitoring projects in general. The specific findings (i.e., the settings) for optimized wood frogs recognizers are unlikely to apply to other species, but the process can be generalized and used to build optimal recognizers for other species.

### 4.1 Increasing training data

We found that increasing training data resulted in only slight improvements to recognizer performance. The most rapid increases in performance were achieved by adding training data from different sites. Adding calls from additional sites into the model could have resulted in small improvements in performance in two ways. First, adding additional sites to the model, especially when the recordings are from the same breeding season (i.e., same year), could help capture variation in wood frog vocalization characteristics by including more unique individuals. The male anuran vocalization contains signals to conspecific females and males that are indicators of competitive ability and fitness, such as size (Giacoma, Cinzia, & Laura, 1997; Howard & Young, 1998) and survivorship (Forsman, Hagman, & Pfenning, 2006), and are subject to sexual selection. In many anuran families, such as ranids (Bee, Perrill, & Owen, 2000) and bufonids (Zweifel, 1968), the dominant frequency of the call is negatively related to the size of the male. Although data on wood frogs specifically are unavailable, it is quite probable that size and age add variability to call characteristics and that training data from different sites could capture more of that variability. Collecting training data from recordings made repeatedly at the same site and calls from within the same recording is increasingly likely to resample the same individuals and therefore have fewer unique individuals represented in the model. This could then overfit the model to the idiosyncrasies of a subset of the variation in wood frog calls and result in poor performance on recognition of calls from individuals not used to build the model (Ginzburg & Jensen, 2004; Radosavljevic & Anderson, 2014) and increase both Type I and II errors. Second, by including additional sites in the model, we include calls that were recorded under different environmental conditions, and this could have a positive effect on the ability of the recognizer to work well in a broader range of conditions (Clement, Murray, Solick, & Gruver, 2014). Some conditions such as wind and rain likely vary as much among recordings as among sites. Other conditions such as the recording unit, the distance of the recorder to the vocalizing individuals, the amount and type of vegetation surrounding and in the wetland, and the amount of anthropogenic sound probably show less variation among recordings than among sites. While research into the causes of this observation would be useful, of greater importance is an evaluation of this relationship with other species of vocalizing anurans to determine whether the weak positive effect on recognizer performance of including calls from many sites is a general rule.

Another explanation for additional training calls being of little value is that wood frogs have a relatively simple call; the optimal feature vector length in Song Scope was four, meaning that the call could be described with four features. More complex anuran, bird, and mammal calls would require more features and could require additional training data to model well. Future research into the relationship between call complexity, variability within and between individuals, and optimal quantities of training data would provide additional insight and guidance for new monitoring programs.

### 4.2 Variable sensitivity analysis

Our objective with the variable sensitivity analysis was to evaluate a method of choosing recognizer variable settings that was reproducible and considers Type I and Type II errors. By having an independent set of recordings upon which to evaluate the different models, we were able to reduce the mean Type I and II errors below that of a recognizer that was created using the trial‐and‐error approach of maximizing fit to the training data.

Maximum syllable gap had more influence on the sensitivity than any other variable. In the Type II error recognizer, this was set at 10 ms. Although this setting was the only one that came close to detecting all wood frog calls at a variety of different chorus sizes, it resulted in an overestimation of the total number of calls as the recognizer made multiple correct hits on different syllables of the same call. This is because wood frog calls are made of 1–4 almost identical syllables. Any recognizer that detects all calls would have to detect the single syllable calls and thus risk making several matches on the multi‐syllable calls. All three other recognizers had overlapping confidence intervals, and the original recognizer had the next best sensitivity of 0.3. Overall, conditional sensitivity changed more in response to adjustments to variable settings than precision. This indicates that researchers may have more control over Type II errors than Type I errors through the use of alternative variable settings. A general limitation with recognizers is the inability to detect overlapping or synchronously recorded calls. As the recognizer works by two‐dimensional spectrogram pattern recognition, calls recorded simultaneously are indistinguishable. Overlapping calls can also be hard to distinguish because the pattern of the vocalization may be altered. As wood frogs are explosive early breeders, they are often observed at high abundances and at least some of the breeding season in some habitats can take place before other species have begun the vocalize. Although the recordings we used to evaluate recognizers contained a gradient in call rates and background noise (the calls of other species as well as environmental noise), high abundances and the associated high call rates present challenges. For example, because fully overlapping calls cannot be counted separately, the ability of the recognizer to count calls as the call rate increases could eventually reach an asymptote if the call rate exceeds the average length of the recognizer model divided by the recording sample length. Therefore, the utility of the recognizer is likely to vary, not only as a function of the spectral properties of the species call, but the intra‐ and interspecific context within which the recordings are made.

Other studies with Song Scope and species recognizers have reported “adjusting” the settings (Waddle et al., 2009), or did not refer to this part of the recognizer design process at all in the methods section (Brauer, Donovan, Mickey, Katz, & Mitchell, 2016; Holmes et al., 2014; Willacy et al., 2015). Under the assumption that wood frogs are not unique in the sensitivity of recognizer performance to variable settings, the field of recognizer development would be advanced by researchers describing what the final recognizer setting were (Brauer et al., 2016; Willacy et al., 2015) and how they arrived at the final recognizer settings (Buxton & Jones, 2012). In an analogous study with species distribution models, Radosavljevic and Anderson (2014) tuned MaxEnt program settings with the goal to minimize overfitting. They discovered that settings 2–4 times higher than the default setting were required to reduce overfitting. In situations such as these where there is no clear intuitive or theoretical way to determine appropriate program settings beforehand, experimentally manipulating the settings and evaluating on new data represent the most likely way of arriving at the optimal choice.

While we are confident the variable sensitivity analysis identified the optimal settings for a wood frog recognizer based on our recording set, it is unknown how general the results are. Had we used a different set of recordings upon which to evaluate Type I errors we could have arrived at different optimal settings for the Type I error recognizer due to differences in background noise. We are also confident that we identified the most sensitive variables, and even if a different set of recordings was used, this list should not change. However, it is unlikely that recognizers for other species of anurans with very different call structures (i.e., Bufonids, Hylids, etc.) will follow the same rank of sensitivities, and they will certainly require different settings of those variables to construct optimal models. Researchers should use the process presented here to fine‐tune their recognizers.

### 4.3 Evaluating recognizer performance

Although transferability is often an implicit objective of ecological models, it is the primary goal of automated call recognizers. Reporting Type I and II error rates for the recordings used to build the recognizer is not a sufficient assessment of the recognizer's performance as they are almost certainly overly optimistic. Using overly optimistic error rates at new sites/regions can bias the interpretation, but the consequences of this bias depend on the project question (occupancy, phenology, or call counts) and the degree of manual verification of the automated results. The challenge in the lack of transferability is seen clearly for Type I errors in this study and others. For example, Clement et al. (2014) evaluated 11 bat species classifiers on new data and observed an increase in the average Type I error rate from 0.06 to 0.29 and 0.17 to 0.32 depending on which library of calls was used to build or test the models. We can conclude that the extra time and effort involved in using independent data to reduce Type I errors are not warranted. In contrast, using independent data to help design recognizers did reduce Type II errors on data from different places and/or times when the emphasis during recognizer development was on minimizing Type II errors. Over all the test sets, the Type II error recognizer had a sensitivity of 0.98 (CI 0.77 – 1.17), compared with all other recognizers at 0.2–0.3. These sensitivity values in excess of 1 are a symptom of the multi‐syllable and variable call structure of the wood frog. While we attempted to penalize variable settings that resulted in sensitivity values greater than 1 at the setting selection stage, we did not alter the results of the evaluation of the recognizer on the training or test datasets to constrain the values below 1.

What causes this lack of transferability in Type I error rate but the reasonable transferability of Type II error rates? It may be that the signal‐to‐noise ratio of the training set was high when compared with the other sets, which indicates better quality recordings in the training set. Also, the background noise of the training data was lower and more variable than the recordings from different places and/or times. It is possible that the optimal variable settings to reduce Type I errors will depend on local sound conditions. On the other hand, choosing settings to reduce Type II errors does not need to consider idiosyncratic background noise in the training set, only the detectability of the frog call itself. If the frog call varies little across space and time, then we should expect less variability in the sensitivity of the recognizer (1‐Type II error rate). There was no clear relationship between distance and time from where the model was developed and where it was used, indicating that variability in recognizer performance is likely a consequence of environmental recording conditions (Buxton & Jones, 2012) which can vary over small spatial scales, rather than variability in wood frog calls per se, which likely only vary over larger spatial scales.

Other factors could affect the transferability of the recognizer. Clement et al. (2014) point out the potential for selection bias to occur when high‐quality calls are manually selected for inclusion into a bat call recognition model. We selected sites and recordings from which to extract individual calls randomly, but we did not choose individual calls randomly. We selected individual calls that had strong signals relative to the background noise and deliberately excluded weak and acoustically contaminated calls. The consequences of this biased selection process could be of the development of recognizers that only detect high‐quality calls. However, if this were true, we would not expect the recognizer designed to avoid Type II errors to have such good performance.

In summary, our data support the conclusions of Clement et al. (2014) that recognizers trained at one place and/or time will rarely be as effective in avoiding Type I errors when used at other places and/or times. However, models designed to reduce Type II error rates were almost as effective at different places and/or times. Researchers should, at minimum, hold some recordings back from use in recognizer creation for use in validation to guard against extreme overfitting (Guthery, Brennan, Peterson, & Lusk, 2005) and obtain estimates of sensitivity/Type II error rates. The type of data held back for validation should be relevant to the objectives of the monitoring program and include sites and times that are of appropriate scales (temporal or geographic) to be a genuine test of out‐of‐sample predictive performance.

### 4.4 Recommendations for recognizer creation

**Figure 10:**
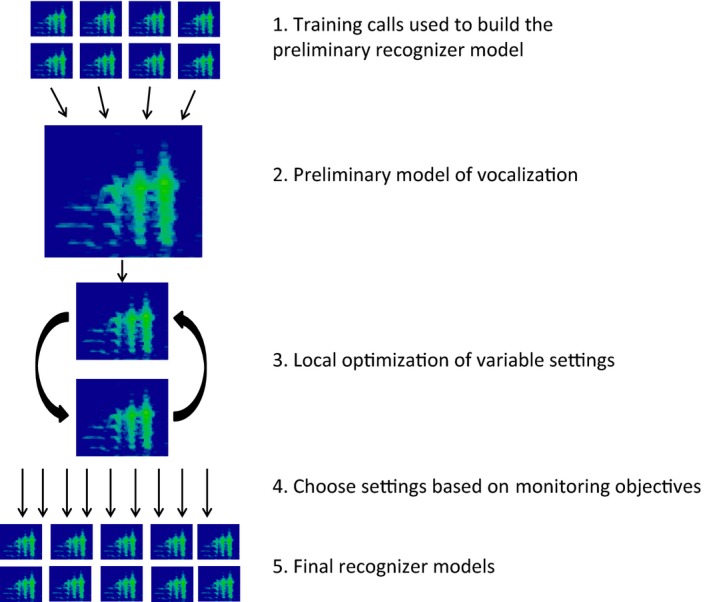
Flowchart showing all the steps in recognizer creation and evaluation

Our results provide a preliminary framework and some recommendations that researchers can use to develop recognizers for wood frogs specifically and, more generally, how our approach might be used for other species. We provide a flowchart to allow readers to visualize the entire process (Figure 10). To summarize the main findings, we found variable settings to be far more important than training data in creating good recognizers. Adding training data from more sites seems to be the most effective way to increase recognizer performance, but again this effect was small when compared to investment in evaluating different variable settings. We showed that considering what recognizer error rates are important and selecting variable settings to match these goals can reduce the rate of false negatives but not the false‐positive rate. Overall, we believe Type I errors are a function of the environmental recording conditions and largely, but not entirely, outside the control of the researcher, whereas Type II errors can be reduced or even eliminated with effort into selecting appropriate variable settings. Extrapolation of Type I error rates for recognizers built on training data from one place and/or time to other places and/or times is probably unjustified under most circumstances.

### 4.5 Future research in automated bioacoustic monitoring

Bioacoustics research is advancing rapidly as improvements to hardware and software are made. Species recognition models represent one of several ways of obtaining information about occupancy, phenology, and relative abundance of a species or species richness at a site. The use of other metrics such as the acoustic complexity index (Pieretti, Farina, & Morri, 2011; Towsey, Wimmer, Williamson, & Roe, 2014), acoustic richness, and dissimilarity index (Depraetere et al., 2012) offer different approaches to the same problem, as do many other machine learning methods (e.g., Gingras & Fitch, 2013). The recent recognition of the problems caused by false‐positive detections and development of occupancy models that incorporate false positives as well as false negatives (McClintock, Bailey, Pollock, & Simons, 2010; Miller et al., 2011) provides a solid link between data collected and analyzed using an automated acoustic platform and occupancy models where uncertainty in parameter estimates can be quantified (Bailey, MacKenzie, & Nichols, 2014). Approaches that compare the quality of methods for obtaining data from recordings are needed to bridge this gap. Finally, our recommendations arise from work done exclusively on wood frogs, and a similar approach should be used on other taxa that are being monitored using automated sound detection to assess how general our conclusions are.

## Conflict of Interest

None declared.
