# Supplementary material for: Designing better frog call recognition models
Source: Ecol Evol. 2017 Mar 30;7(9):3087–99. doi: 10.1002/ece3.2730 (PMC5415519; doi:10.1002/ece3.2730)
Supplement: Supplementary file 1 [file ECE3-7-3087-s001.docx]

|  | |  | | | | **TARGETED** | | | |  | | | | **ACHIEVED** | |  | | |  | | | |  | | | | |
| --- | --- | --- | --- | --- | --- | --- | --- | --- | --- | --- | --- | --- | --- | --- | --- | --- | --- | --- | --- | --- | --- | --- | --- | --- | --- | --- | --- |
| **Type** | **Recognizer ID** | | **sites** | **recordings / site** | | | | **calls / recordings** | | | **training calls** | **sites** | **recordings / site** | | **calls / recordings** | | | **training calls** | **AUROCC** | | **Y.J** | | | | **Prec.** | | **Sens.** |
| site | 0.05 | | 1 | 6 | | | | 1 | | | 6 | 1 | 4 | | 1 | | | 4 | 0.692 | | 52.36 | | | | 0.809 | | 0.432 |
| site | 0.1 | | 1 | 12 | | | | 1 | | | 12 | 1 | 8 | | 1 | | | 8 | 0.674 | | 53.13 | | | | 0.811 | | 0.415 |
| site | 0.2 | | 1 | 15 | | | | 1 | | | 15 | 1 | 12 | | 1.3 | | | 16 | 0.689 | | 54.78 | | | | 0.827 | | 0.402 |
| site | 0.3 | | 1 | 15 | | | | 3 | | | 45 | 1 | 13 | | 2.5 | | | 32 | 0.703 | | 54.35 | | | | 0.817 | | 0.445 |
| site | 0.4 | | 1 | 15 | | | | 5 | | | 75 | 1 | 15 | | 4.3 | | | 65 | 0.672 | | 55.84 | | | | 0.808 | | 0.380 |
| site | 0.5 | | 1 | 15 | | | | 12 | | | 180 | 1 | 15 | | 8.5 | | | 128 | 0.702 | | 59.57 | | | | 0.804 | | 0.389 |
| site | 1 | | 2 | 15 | | | | 12 | | | 360 | 2 | 15 | | 14.4 | | | 431 | 0.715 | | 62.76 | | | | 0.823 | | 0.469 |
| site | 2 | | 5 | 15 | | | | 12 | | | 900 | 5 | 14 | | 13.5 | | | 943 | 0.759 | | 64.41 | | | | 0.835 | | 0.459 |
| site | 3 | | 8 | 15 | | | | 12 | | | 1440 | 8 | 14.4 | | 11 | | | 1270 | 0.778 | | 64.15 | | | | 0.838 | | 0.467 |
| site | 4 | | 11 | 15 | | | | 12 | | | 1980 | 11 | 13.6 | | 11 | | | 1667 | 0.785 | | 65.45 | | | | 0.835 | | 0.435 |
| site | 5 | | 14 | 15 | | | | 12 | | | 2520 | 14 | 12.6 | | 10.3 | | | 2076 | 0.782 | | 68.98 | | | | 0.829 | | 0.478 |
| site | 6 | | 17 | 15 | | | | 12 | | | 3060 | 17 | 13.8 | | 10.3 | | | 2576 | 0.777 | | 67.07 | | | | 0.833 | | 0.459 |
| site | 7 | | 20 | 15 | | | | 12 | | | 3600 | 20 | 13.4 | | 10.5 | | | 3074 | 0.774 | | 66.86 | | | | 0.831 | | 0.518 |
| site | 8 | | 23 | 15 | | | | 12 | | | 4140 | 25 | 13.8 | | 10.3 | | | 3564 | 0.759 | | 67.67 | | | | 0.839 | | 0.446 |
| site | 9 | | 25 | 15 | | | | 12 | | | 4500 | 27 | 13 | | 10.4 | | | 3643 | 0.792 | | 65.38 | | | | 0.845 | | 0.503 |
| site | 10 | | 28 | 15 | | | | 12 | | | 5040 | 28 | 13.5 | | 10.3 | | | 4080 | 0.776 | | 68.28 | | | | 0.830 | | 0.457 |
| recording | 10.5 | | 28 | 1 | | | | 12 | | | 336 | 28 | 1 | | 10.1 | | | 283 | 0.773 | | 64.65 | | | | 0.839 | | 0.469 |
| recording | 11 | | 28 | 4 | | | | 12 | | | 1344 | 28 | 1.9 | | 10.8 | | | 605 | 0.778 | | 67.74 | | | | 0.837 | | 0.449 |
| recording | 12 | | 28 | 2 | | | | 12 | | | 672 | 28 | 3.6 | | 10.1 | | | 1094 | 0.760 | | 68.34 | | | | 0.853 | | 0.450 |
| recording | 13 | | 28 | 6 | | | | 12 | | | 2016 | 28 | 5.6 | | 10.1 | | | 1694 | 0.773 | | 66.77 | | | | 0.830 | | 0.485 |
| recording | 14 | | 28 | 8 | | | | 12 | | | 2688 | 28 | 7.6 | | 10.1 | | | 2304 | 0.782 | | 68.28 | | | | 0.839 | | 0.473 |
| recording | 15 | | 28 | 9 | | | | 12 | | | 3024 | 28 | 8.2 | | 10.1 | | | 2488 | 0.788 | | 67.29 | | | | 0.842 | | 0.454 |
| recording | 16 | | 28 | 10 | | | | 12 | | | 3360 | 28 | 9.1 | | 10.1 | | | 2763 | 0.788 | | 65.73 | | | | 0.856 | | 0.479 |
| recording | 17 | | 28 | 11 | | | | 12 | | | 3696 | 28 | 9.8 | | 10.4 | | | 3033 | 0.788 | | 67.61 | | | | 0.838 | | 0.458 |
| recording | 18 | | 28 | 13 | | | | 12 | | | 4368 | 28 | 11.5 | | 10.3 | | | 3560 | 0.782 | | 68.39 | | | | 0.841 | | 0.463 |
| recording | 19 | | 28 | 14 | | | | 12 | | | 4704 | 28 | 12.3 | | 10.4 | | | 3822 | 0.785 | | 67.69 | | | | 0.852 | | 0.454 |
| recording | 20 | | 28 | 15 | | | | 12 | | | 5040 | 28 | 13.5 | | 10.3 | | | 4080 | 0.776 | | 68.28 | | | | 0.830 | | 0.457 |
|  |  | | **TARGETED** | | | | | | | | | **ACHIEVED** | | | | | | |  | |  | | | |  | |  |
|  |  | |  | | | | | | | | |  | | | | | | |  | |  | | | |  | |  |
| **Type** | **Recognizer ID** | | **sites** | **recordings / site** | | | | **calls / recordings** | | | **training calls** | **sites** | **recordings / site** | | **calls / recordings** | | | **training calls** | **AUROCC** | | **Y.J** | | | **Prec.** | | **Sens.** | |
| calls | 20.08 | | 6 | 1 | | | | 1 | | | 6 | 6 | 1 | | 1 | | | 6 | 0.709 | | 58.83 | | | 0.819 | | 0.456 | |
| calls | 20.09 | | 12 | 1 | | | | 1 | | | 12 | 12 | 1 | | 1 | | | 12 | 0.729 | | 64.99 | | | 0.818 | | 0.515 | |
| calls | 20.1 | | 25 | 1 | | | | 1 | | | 25 | 23 | 1.04 | | 1 | | | 25 | 0.749 | | 65.31 | | | 0.840 | | 0.473 | |
| calls | 20.125 | | 27 | 2 | | | | 1 | | | 54 | 27 | 1.8 | | 1 | | | 49 | 0.703 | | 67.66 | | | 0.822 | | 0.437 | |
| calls | 20.25 | | 28 | 4 | | | | 1 | | | 112 | 28 | 3.5 | | 1 | | | 98 | 0.761 | | 66.21 | | | 0.838 | | 0.492 | |
| calls | 20.5 | | 28 | 8 | | | | 1 | | | 210 | 28 | 7 | | 1 | | | 196 | 0.759 | | 67.44 | | | 0.844 | | 0.478 | |
| calls | 21 | | 28 | 15 | | | | 1 | | | 420 | 28 | 13.8 | | 1 | | | 392 | 0.758 | | 69.43 | | | 0.835 | | 0.466 | |
| calls | 22 | | 28 | 15 | | | | 2 | | | 840 | 28 | 12.9 | | 2 | | | 768 | 0.738 | | 66.95 | | | 0.835 | | 0.469 | |
| calls | 23 | | 28 | 15 | | | | 4 | | | 1680 | 28 | 13 | | 3.8 | | | 1476 | 0.761 | | 66.33 | | | 0.826 | | 0.501 | |
| calls | 24 | | 28 | 15 | | | | 5 | | | 2100 | 28 | 13.1 | | 4.7 | | | 1845 | 0.748 | | 68.25 | | | 0.845 | | 0.473 | |
| calls | 25 | | 28 | 15 | | | | 6 | | | 2520 | 28 | 13.1 | | 5.6 | | | 2219 | 0.756 | | 65.14 | | | 0.837 | | 0.503 | |
| calls | 26 | | 28 | 15 | | | | 7 | | | 2940 | 28 | 13.1 | | 6.5 | | | 2578 | 0.776 | | 66.42 | | | 0.847 | | 0.470 | |
| calls | 27 | | 28 | 15 | | | | 8 | | | 3360 | 28 | 13.1 | | 7 | | | 2753 | 0.758 | | 68.41 | | | 0.832 | | 0.458 | |
| calls | 28 | | 28 | 15 | | | | 10 | | | 4200 | 28 | 13.1 | | 8.5 | | | 3348 | 0.779 | | 67.45 | | | 0.853 | | 0.451 | |
| calls | 29 | | 28 | 15 | | | | 11 | | | 4620 | 28 | 13.2 | | 9.5 | | | 3715 | 0.772 | | 68.16 | | | 0.831 | | 0.453 | |
| calls | 30 | | 28 | 15 | | | | 12 | | | 5040 | 28 | 13.5 | | 10.3 | | | 4080 | 0.776 | | 68.28 | | | 0.830 | | 0.457 | |
|  |  | |  | |  | |  | |  | | |  | |  |  | |  | |  |  | |  | | | |  | |

Appendix 1. Experimental design and results of increasing the total and type of training data on recognizer performance. The 3 types of increasing training data are sites (among-site), recordings (among-recordings), and calls (within-recordings). Recognizer ID’s 1-10, 11-20, and 21-30 are the initial 30 models and the other IDs (with decimal places) are the 13 follow-up models. The “targeted” and “achieved” columns show the total and type of training data that was attempted and what was actually achieved. The recordings per site and calls per recording are averages. AUROCC is the area under the receiver operating characteristic curve, Y.J. is the optimal Youden’s J score threshold, Prec. and Sens. are the conditional precision and sensitivity recognizer performance metrics.
