# Supplementary material for: Designing better frog call recognition models
Source: Ecol Evol. 2017 Mar 30;7(9):3087–99. doi: 10.1002/ece3.2730 (PMC5415519; doi:10.1002/ece3.2730)
Supplement: Supplementary file 2 [file ECE3-7-3087-s002.docx]

Appendix 2. Song Scope V.4.1.3 A variables used in recognizer development and the number of different settings evaluated in the variable sensitivity analysis

| **Variable** | **Range** | **Number of possible states** | **Description** | **Number of Models Evaluated** |
| --- | --- | --- | --- | --- |
| Maximum  complexity | 16 -- 48 | 32 | Also known as “model states”; controls the maximum number of different “versions” of call the recognizer can model. | 9 |
| Maximum  resolution | 4 -- 32 | 28 | Also known as the “feature vector length”; controls the maximum number of dimensions that can be modelled. | 8 |
| FFT size | 32 -- 4096 | 8 | Controls the spectrogram by adjusting the spectral and temporal bin size through the Fast Fourier Transform algorithm. | 8 |
| FFT overlap | 0 -- 7/8 | 4 | Controls the amount of overlap between the spectrogram bins. | 4 |
| Dynamic  range | 10 -- 90 | 80 | Limits the decibel range of signals that will be detected when compared to the strongest signal. | 9 |
| Background  filter | 0 -- 5s | 6 | Sets the amount of time used to calculate the average background noise and then remove it from the spectrogram. | 6 |
| Maximum  syllable length | 1 -- 2000 | 2000 | Sets the maximum length (in time) of a syllable in the vocalization of interest. | 10 |
| Maximum syllable gap | 1 -- 2000 | 2000 | Sets the maximum gap (in time) between two syllables in the vocalization of interest | 10 |
| Maximum song length | 1 -- 60000 | 60000 | Sets the maximum length (in time) of the complete vocalization. | 11 |
