# Supplementary material for: Designing better frog call recognition models
Source: Ecol Evol. 2017 Mar 30;7(9):3087–99. doi: 10.1002/ece3.2730 (PMC5415519; doi:10.1002/ece3.2730)
Supplement: Supplementary file 3 [file ECE3-7-3087-s003.docx]

Appendix 3. Table shows the variable settings and Song Scope performance statistics for the 3 final recognizer models (built using the iterative optimization approach) and the original model (built using the trial and error approach).

|  | Recognizer Models | | | |
| --- | --- | --- | --- | --- |
| **Variable** | **1--1** | **5--1** | **1--5** | **Original** |
| Max complexity | 32 | 28 | 32 | 32 |
| Max resolution | 4 | 4 | 4 | 10 |
| FFT size | 1024 | 1024 | 256 | 256 |
| FFT overlap | 0.5 | 0.5 | 0.5 | 0.5 |
| Background filter | 2 | 1 | 2 | 1 |
| Dynamic range | 20 | 20 | 20 | 25 |
| Max syllable (ms) | 260 | 300 | 260 | 56 |
| Max syllable gap (ms) | 50 | 290 | 10 | 56 |
| Max song (ms) | 5000 | 5000 | 5000 | 200 |
| Frequency min | 36 | 36 | 9 | 9 |
| Frequency range | 112 | 112 | 28 | 30 |
|  |  |  |  |  |
| Cross Training (%) | 72.85 ± 11.06 | 72.77 ± 8.59 | 73.52 ± 12.6 | 66.37 ± 10.81 |
| Total Training (%) | 73.07 ± 10.72 | 72.55 ± 8.94 | 73.29 ± 14.23 | 66.45 ± 11.26 |
| Model States | 30 | 27 | 30 | 30 |
| State Usage | 3 ± 2 | 3 ± 2 | 5 ± 4 | 7 ± 5 |
| Feature Vector | 4 | 4 | 4 | 10 |
| Mean Symbols | 4 ± 3 | 4 ± 3 | 7 ± 7 | 11 ± 9 |
| Syllable Types | 8 | 7 | 5 | 5 |
| Mean Duration (s) | 0.19 ± 0.1 | 0.19 ± 0.1 | 0.08 ± 0.07 | 0.13 ± 0.1 |
